# Correlation Analysis of Huayu Tongmai Decoction Intervention and Prognosis Indexes of Patients with Carotid Atherosclerosis

**DOI:** 10.1155/2021/2739092

**Published:** 2021-10-31

**Authors:** Guangqing Cheng, Xiaoni Yan, Fengmeng Wang, Chao Chen

**Affiliations:** ^1^Department of Gerontology, The First Affiliated Hospital of Shandong First Medical University & Shandong Provincial Qianfoshan Hospital, Jinan 250014, Shandong, China; ^2^Department of Rehabilitation, Zibo TCM-Integrated Hospital, Zibo 255026, Shandong, China

## Abstract

**Background:**

Carotid atherosclerosis (CAS) is a common disease which seriously threatens the health of senile patients. The studies have indicated that traditional Chinese medicine (TCM) may effectively improve the symptom of CAS, while the therapeutic effect of Huayu Tongmai decoction on CAS remains unclear. Thus, this study aimed to explore the correlation between traditional Chinese medicine Huayu Tongmai decoction intervention and prognosis indexes of patients with CAS.

**Methods:**

Ninety CAS patients admitted to *Zibo TCM-Integrated Hospital* from September 2018 to September 2020 were selected as the research object and randomly divided into the control group and the observation group according to the male-female ratio of 1 : 1. Patients in the control group accepted the atorvastatin intervention, and on this basis, patients in the observation group were further intervened with TCM Huayu Tongmai decoction. Before and after treatment, patients' levels of total cholesterol (TC), triglyceride (TG), and low-density lipoprotein cholesterol (LDL-C) were measured by the enzyme photometric colorimetry; hypersensitive c-reactive protein (hs-CRP) levels were measured by the ELISA method; nitric oxide (NO) levels were measured by the nitrate reductase assay and endothelin-1 (ET-1) levels were measured by radioimmunoassay; and the right and left carotid internal diameter (CAD), intima-media thickness (IMT), and plaque volume were measured by carotid ultrasonography.

**Results:**

The TC, TG, and LDL-C levels significantly decreased in patients compared to those before intervention; compared with the control group, patients who accepted Huayu Tongmai decoction combined with atorvastatin saw more significant improvement in their blood lipid indexes (*P* < 0.01); after intervention, patients' hs-CRP and ET-1 levels dropped significantly while the NO level rose remarkably, and between the two groups, the improvement in levels of hs-CRP, ET-1, and NO of patients in the observation group was significantly better (*P* < 0.01); it was concluded from the imaging diagnosis results that compared with using atorvastatin alone, the combined intervention could better improve patients' CAD, IMT, and plaque volume.

**Conclusion:**

Huayu Tongmai decoction can effectively improve patients' blood lipid, reduce inflammatory response, enhance levels of relevant regulatory factors of CAS, and alleviate the symptoms.

## 1. Introduction

Atherosclerosis (AS) has a high incidence rate in the middle-aged and elderly people and is a direct cause of organ insufficiency as well as a major factor triggering coronary heart disease (CHD), cerebral ischemia, and peripheral vascular diseases in patients [1, 2]. Currently, the main therapeutic strategies for AS in clinic include surgical intervention and drug intervention. Surgical intervention is the major surgical means to ameliorate the symptoms of AS in patients, with the characteristics such as instant effect and good treatment outcome [3]. But AS patients are mostly elderly, so the risk of surgical intervention is usually higher and the surgical conditions have some limitations on their physical fitness. In response to this, statins such as atorvastatin or simvastatin are often used clinically to delay the progression of AS in patients. In addition, statins can inhibit the progression of carotid atherosclerosis (CAS) by reducing blood lipid levels, reducing body TG synthesis, and improving cholesterol levels in patients [4]. However, despite atorvastatin's ability to effectively reduce patients' blood lipids, some studies have confirmed that the drug may trigger adverse effects such as headache, edema, and diarrhea [5].

In recent years, the value of traditional Chinese medicine (TCM) in the treatment of CAS has received extensive attention. Studies have confirmed that active ingredients from some herbs in TCM have good therapeutic effects on AS [6]. TCM has proved that the Huayu Tongmai decoction, which contains tangshen, danshen root, golden thread, Sichuan lovage rhizome, hawthorn fruit, tall gastrodia tuber, coix seed, turmeric root tuber, and other herbs, has the effect of ameliorating inflammation and activating blood circulation to eliminate stasis, among which danshen root, golden thread, and Sichuan lovage rhizome have documented efficacy of improving blood lipid levels in patients. This study focused on the therapeutic effect of Huayu Tongmai decoction on CAS and aimed to provide a certain reference for the study of TCM on ameliorating CAS.

## 2. Study Methods

### 2.1. General Information

**Figure 1:**
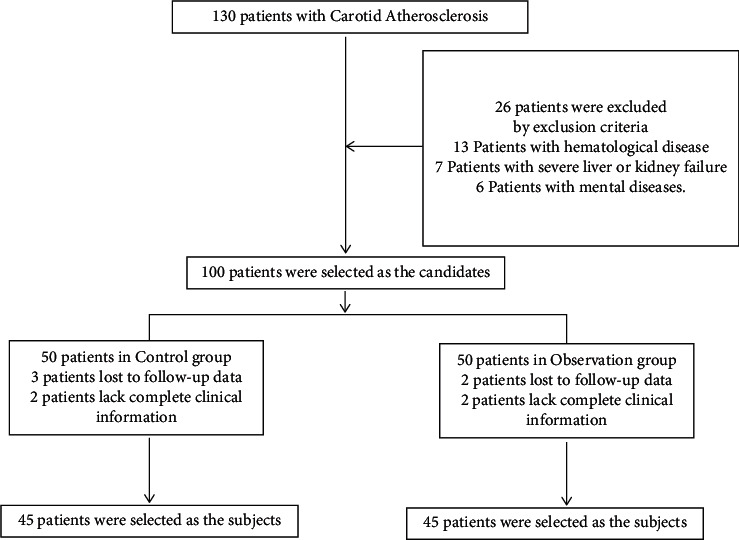
The patient flow of the study.

The study was approved by the ethics committee of *Zibo TCM-Integrated Hospital*, and all experiments were conducted in accordance with the Declaration of Helsinki (as revised in 2013). Ninety CAS patients treated in *Zibo TCM-Integrated Hospital* from September 2018 to September 2020 were selected as the research object and randomly divided into the control group and the observation group according to the male-female ratio of 1 : 1 (Figure 1). As shown in Table 1, patients' age, gender, body mass index (BMI), drinking history, smoking history, and place of residence were not significantly different between the two groups. This study was a randomized controlled trial.

### 2.2. Inclusion and Exclusion Criteria

1. *Inclusion Criteria*. Patients' general clinical information were complete; patients were diagnosed with AS after imaging examination; patients met the diagnosis standards for CAS; patients did not use any drugs that affect their blood lipid or coagulation function in the recent 3 months before the study; and patients or their accompanying family members signed the informed consent.
2. *Exclusion Criteria*. Patients had liver and kidney failure; patients suffered from hematological diseases that would seriously affect the experiment results; patients had serious mental diseases; and patients could not accept clinical follow-up.

### 2.3. Methods

1. Patients in the control group accepted the conventional western medicine treatment, i.e., orally taking 100 mg of aspirin enteric-coated tablets (Beijing Shuguang Pharmaceutical Co., Ltd., Beijing, China) and 10 mg of atorvastatin (Beijing Jialin Pharmaceutical Co., Ltd., Beijing, China) every day for 3 months.
2. On the basis of drug administration of the control group, patients in the observation group took the concentrate granules of Huayu Tongmai formula. The drug was mixed with 200 mL of boiling water and split into two doses, with one in the morning and one in the evening. The Huayu Tongmai formula (provided by Sanjiu Medical & Pharmaceutical Co. Ltd.) contained tangshen, danshen root, and coix seed (20 g each), Sichuan lovage rhizome, hawthorn fruit, tall gastrodia tuber, and turmeric root tuber (10 g each), and 6 g of golden thread. The patients received the intervention of Huayu Tongmai decoction for 3 months.

### 2.4. Observation Indexes

1. *Blood Lipid Measurement*. Before and after intervention, 3 mL of fasting venous blood was drawn from patients in the morning, put into the stimulative coagulation tube that contained separation gel, and placed under room temperature for 1 hour. Then, the venous blood was centrifuged under 3,000 r/min for 10 min to extract the supernatant, which was then placed in the freezer of −20°C for reservation. The levels of triglyceride (TG), total cholesterol (TC), and low-density lipoprotein cholesterol (LDL-C) were measured by automatic biochemical analysis.
2. *Carotid Ultrasonography Diagnosis*. Under the probe frequency of 10.5 MHz, patients' right and left common carotid arteries were detected for the carotid internal diameter (CAD), intima-media thickness (IMT), and plaque volume with the U.S. ATL. HDL-5000 color doppler ultrasound machine.
3. *Hypersensitive C-Reactive Protein (hs-CRP) Level Detection*. 3 mL of fasting venous blood was drawn from patients in the morning, put into the stimulative coagulation tube that contained separation gel, and placed under room temperature for 1 hour. The venous blood was centrifuged under 3,000 r/min to extract the supernatant, which was then placed in the freezer of −20°C for reservation. The levels of hs-CRP in the serum were measured with the ELISA kit (Shanghai Meilian Biological Co., Ltd., Shanghai, China).
4. *Vascular Endothelial Function Detection*. Before and after treatment, 3 mL of venous blood was drawn from the patients, put into the vacuum sampling tube and placed under room temperature for 30 minutes, and then it was centrifuged under 3,500 r/min for 10 minutes to extract the supernatant, which was then placed under −20°C for reservation. The level of nitric oxide (NO) was measured by the nitrate reductase assay and the level of endothelin-1 (ET-1) was measured by the radioimmunoassay.

### 2.5. Statistical Processing

The results of the experiment were analyzed by SPSS19.0, the between-group or within-group differences were examined with *t* test or analyzed by ANOVA and Tukey test and recorded as mean value ± standard deviation, and differences were considered statistically significant at *P* < 0.05.

## 3. Results

### 3.1. Changes in Levels of TC, TG, and LDL-C in Patients of the Two Groups before and after Intervention

The levels of TC, TG, and LDC-C in patients of the two groups before and after drug intervention are shown in Table 2. The results indicated that after intervention, the levels of TC, TG, and LDC-C in patients of the two groups significantly decreased (*P* < 0.05), and these levels were significantly lower in the observation group than in the control group (*P* < 0.05).

### 3.2. Changes in hs-CRP Levels of Patients in the Two Groups before and after Intervention

**Figure 2:**
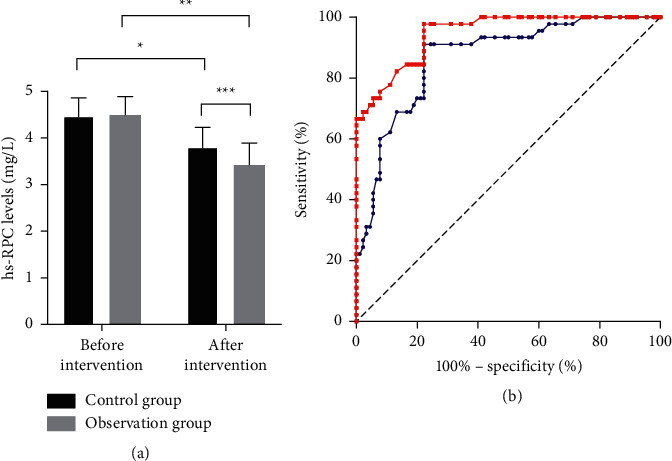
Changes in patients' hs-CRP levels before and after intervention. (a) Patients' hs-CRP levels before and after intervention. (b) ROC curves of changes in patients' hs-CRP levels after treatment (blue: control group; red: observation group). ^*∗*^ and ^*∗∗*^, respectively, indicated the comparison within the control group and that within the observation group before and after intervention, and ^*∗∗∗*^ indicated the comparison between the control group and the observation group after intervention.

The hs-CRP levels in patients of the two groups before and after drug intervention are shown in Figure 2. The results indicated that after intervention, the serum hs-CRP levels of both groups significantly decreased (*P* < 0.05), and compared with the control group, the observation group achieved significantly lower serum hs-CRP levels (*P* < 0.05) and more obvious improvement (*P* < 0.05).

### 3.3. Changes in NO Levels of Patients in the Two Groups before and after Intervention

**Figure 3:**
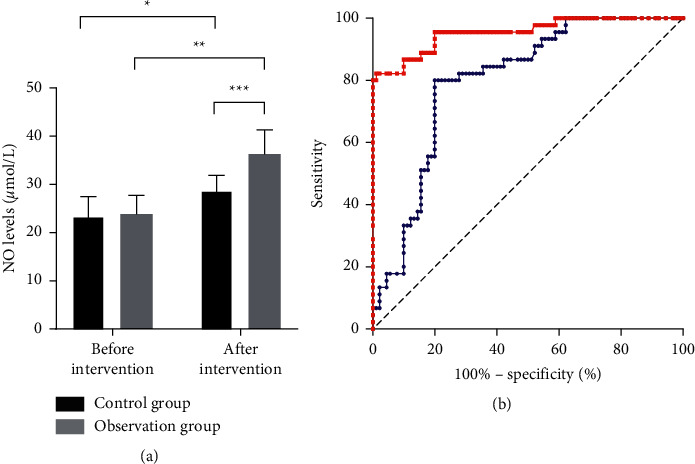
Changes in patients' NO levels before and after intervention. (a) Patients' NO levels before and after intervention. (b) ROC curves of changes in patients' NO levels after treatment (blue: control group; red: observation group). ^*∗*^ and ^*∗∗*^, respectively, indicated the comparison within the control group and that within the observation group before and after intervention, and ^*∗∗∗*^ indicated the comparison between the control group and the observation group after intervention.

The serum NO levels in patients of the two groups before and after drug intervention are shown in Figure 3. The results indicated that after intervention, the NO levels of both groups significantly increased (*P* < 0.05), and compared with the control group, the observation group achieved significantly higher serum NO levels (*P* < 0.05) and more obvious improvement (*P* < 0.05).

### 3.4. Changes in ET-1 Levels of Patients in the Two Groups before and after Intervention

**Figure 4:**
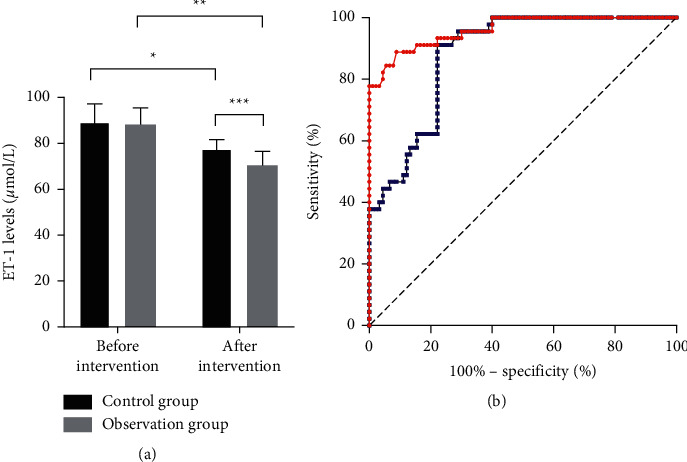
Changes in patients' ET-1 levels before and after intervention. (a) Patients' ET-1 levels before and after intervention. (b) ROC curves of changes in patients' ET-1 levels after treatment (blue: control group; red: observation group). ^*∗*^ and ^*∗∗*^, respectively, indicated the comparison within the control group and that within the observation group before and after intervention, and ^*∗∗∗*^ indicated the comparison between the control group and the observation group after intervention.

The serum ET-1 levels in patients of the two groups before and after drug intervention are shown in Figure 4. The results indicated that after intervention, the ET-1 levels of both groups dropped significantly (*P* < 0.05), and compared with the control group, the observation group achieved significantly lower serum ET-1 levels (*P* < 0.05) and more obvious improvement (*P* < 0.05).

### 3.5. Ultrasonic Diagnostic Examination

The values of CAD, IMT, and plaque volume of the patients in the two groups before and after drug intervention are shown in Table 3. The results indicated that after intervention, the values of CAD, IMT, and plaque volume of both groups were improved significantly (*P* < 0.05), and compared with the control group, the observation group achieved significantly lower values of CAD, IMT, and plaque volume (*P* < 0.05) and more obvious improvement (*P* < 0.05) (see Table 3).

## 4. Discussion

CAS plaque is one of the leading causes of ischemic stroke in patients. Currently, the therapies for CAS mainly include endovascular treatment and drug intervention, of which the endovascular treatment is able to improve arterial blood flow in patients through arterial thrombolysis, endovascular thrombus removal, and implantation of stents [7, 8]. However, there are certain risks of intravascularly implanting stents because it may cause damage to patients' blood vessel wall and also trigger new branch artery occlusion and then worsen the condition. Therefore, drug intervention is often used clinically to control the condition of AS patients [9]. Statins such as atorvastatin serve as an important class of drugs to prevent AS and intervene in the course of CAS in clinic [10]. Recent studies have confirmed that some TCM strategies work well in the treatment of CAS [11]. Based on this, the study explored the effect of combining Huayu Tongmai decoction with atorvastatin in the improvement of symptoms in CAS patients and confirmed that after intervention, the levels of TC, TG, and LDL-C in patients who accepted the combined therapy were significantly lower than those who received atorvastatin alone, indicating that Huayu Tongmai decoction could effectively improve the patients' blood lipid levels and then delay the progression of CAS. Huayu Tongmai decoction contained tangshen, danshen root, golden thread, Sichuan lovage rhizome, hawthorn fruit, tall gastrodia tuber, coix seed, and turmeric root tuber, of which danshen root and golden thread had the effect of improving the levels of TC, TG, and LDL-C in human body [12–14] because danshen root could regulate the OTUD7B/KLF4/NMHC IIA signal axes to improve the abnormal proliferation of vascular smooth muscle cells and vascular remodeling [15], which had been proved by Yang et al., and golden thread also had been shown to alleviate CAS symptoms in rats [16]. In addition, according to some studies, Sichuan lovage rhizome could stimulate the NO synthase expression of vascular endothelial cells in rats and then improve the NO levels and had significant anti-inflammation effect as well [17].

There is a close relationship between the formation and development of AS and the levels of inflammatory factors in the body of patients because high levels of inflammatory factors can induce endothelial cell damage. CRP may serve as a risk marker for CAS, which may promote endothelial cell inflammation and injury by mediating the upregulation of adhesion molecules and chemotactic factors, and studies have confirmed that CRP levels significantly increase in the lesions of AS patients [18]. The study herein proved that applying Huayu Tongmai decoction on the basis of atorvastatin intervention could further improve the hs-CRP levels in patients. In the research by Luo et al., it was found that danshen root could effectively lower the hs-CRP levels in CHD patients and then improve their systemic inflammatory levels [19]. Xue et al. also proved that golden thread could significantly lower the hs-CRP levels in rats to remarkably ameliorate the symptoms of carotid thrombosis [16]. Endothelial cells have certain regulatory effect on the contraction and relaxation of blood vessels, and normal endothelial cells can effectively inhibit the formation of arterial plaques by suppressing the adhesion and stacking of platelets. Vascular endothelial cells dysfunction is one of the main reasons for initiating AS, and platelets are able to aggregate at damaged endothelial cells, which leads to thrombosis [20, 21]. Studies have confirmed that NO can effectively inhibit the aggregation of inflammatory factors and platelets and suppress free radical induced endothelial cell injury through antagonism. In the study herein, compared with single atorvastatin intervention, patients who accepted the combined intervention of Huayu Tongmai decoction and atorvastatin saw obvious increase in their serum NO levels. Zhang et al. confirmed in their study that using danshen root and hawthorn fruit together could significantly lower the levels of TC, TG, and LDL-C in patients, greatly reduce the levels of IL-1*β* and IL-18 to improve the systematic inflammation, and inhibit endothelin expression by increasing the serum NO level to improve the CAS symptoms in patients [22]. ET-1 can regulate vasoconstriction, but overexpressed ET-1 is able to promote the progression of AS. Studies have shown that the expression abundance of ET-1 can reach about twice the normal level at sites of coronary atherosclerosis [23]. Our study demonstrated that Huayu Tongmai decoction could further reduce the ET-1 levels in patients' serum on the basis of atorvastatin. Wu et al. confirmed that Sichuan lovage rhizome could effectively reduce the ET-1 level and improve the blood supply volume to the brain in a migraine rat model [17]. In addition, the results of imaging diagnosis herein showed that patients who received the combined intervention obtained more significant improvements in CAD, IMT, and plaque volume than those who received single atorvastatin intervention, indicating that the adoption of Huayu Tongmai decoction on the basis of atorvastatin could effectively improve the symptoms of CAS in patients. Further investigation is required on the conclusion of the study due to the limited number of the patients selected. In addition, the mechanism of Huayu Tongmai decoction in treating AS should also be further studied in depth.

In conclusion, the study proves that Huayu Tongmai decoction can significantly improve the symptoms of CAS in patients, which is worthy of application in clinic. Moreover, this study only focused on the therapeutic effect of Huayu Tongmai decoction on CAS, and thus the pharmacological mechanism of Huayu Tongmai decoction in progression of improving correlation analysis is necessary.

## Figures and Tables

**Table 1:** Patients' general information.

Item	Control group	Observation group	*P*	*t*
Mean age	67.16 ± 7.73	67.07 ± 7.61	*P* > 0.05	0.056
Male-female ratio				
Male	23	21	*P* > 0.05	0.098
Female	22	24		
BMI (kg/m^2^)	23.23 ± 5.23	23.36 ± 5.09	*P* > 0.05	0.119
*Smoking history*				
Yes	15	16	*P* > 0.05	0.445
No	40	39		
*Drinking history*				
Yes	16	18	*P* > 0.05	0.17
No	39	37		
*Place of residence*				
Urban area	29	27	*P* > 0.05	0.189
Rural area	16	18		

**Table 2:** Changes in levels of TC, TG, and LDL-C in patients of the two groups before and after intervention.

Group	Time	TG (mmoL/L)	TC (mmoL/L)	LDL-C (mmoL/L)
Control group (*n* = 45)	Before	1.86 ± 0.39	5.67 ± 0.79	3.53 ± 0.63
	After	1.53 ± 0.21^a^	4.13 ± 0.63^a^	2.69 ± 0.32^a^

Observation group (*n* = 45)	Before	1.85 ± 0.32	5.69 ± 0.71	3.51 ± 0.53
	After	1.25 ± 0.17^ab^	3.81 ± 0.57^ab^	2.49 ± 0.39^ab^

*Note*. a indicated significant within-group differences in patients' indexes before and after intervention (*P* < 0.05), and b indicated significant between-group differences in patients' indexes before and after intervention (*P* < 0.05).

**Table 3:** Results of common carotid artery ultrasonography before and after intervention.

Group	Time	CAD (mm)	IMT (mm)	Plaque volume (mm^3^)
Control group (*n* = 45)	Before	5.79 ± 0.53	1.28 ± 0.23	89.51 ± 8.93
	After	4.96 ± 0.39^a^	0.98 ± 0.19^a^	46.27 ± 3.23^a^

Observation group (*n* = 45)	Before	5.83 ± 0.60	1.30 ± 0.21	89.51 ± 9.13
	After	4.70 ± 0.37^ab^	0.91 ± 0.17^ab^	43.39 ± 3.09^ab^

*Note*. a indicated significant within-group differences in patients' indexes before and after intervention (*P* < 0.05), and b indicated significant between-group differences in patients' indexes before and after intervention (*P* < 0.05).

## Data Availability

The data used to support the findings of this study are available on reasonable request from the corresponding author.
